# The Effects of Aldosterone on Hypertension-Associated Kidney Injury in a Tg-hAS Mouse Model

**DOI:** 10.3390/biology13121084

**Published:** 2024-12-22

**Authors:** Huiying Gu, Zhe Chen, Nicole Du, Sisi Yang, Yongqi Yu, Yansheng Du

**Affiliations:** 1 Department of Neurology, Indiana University School of Medicine, Indianapolis, IN 46202, USA; huiygu@iu.edu (H.G.); zhepi2006@163.com (Z.C.); tjh_yss@tjh.tjmu.edu.cn (S.Y.); yoyu@iu.edu (Y.Y.); 2 Boston Children’s Hospital, Boston, MA 02115, USA; nicole.du@childrens.harvard.edu

**Keywords:** aldosterone, aldosterone synthase, hypertension, FAD286, spironolactone, kidney injury, macrophage, collagen IV

## Abstract

Hypertension remains a global health challenge due to its high prevalence and association with premature morbidity and mortality. Aldosterone, a mineralocorticoid hormone, and its receptor, the mineralocorticoid receptor (MR), are highly implicated in hypertension pathogenesis. Aldosterone synthase is the sole enzyme responsible for producing aldosterone in humans. We established transgenic mice carrying the human aldosterone synthase gene (cyp11B2) and showed dramatically increased levels of aldosterone in female hemizygotes. High-salt diets persistently increased blood pressure in these mice, and salt-induced hypertension was significantly ameliorated by reducing aldosterone levels via an aldosterone synthase inhibitor or blocking MR via an MR inhibitor. Since both hypertension and hyperaldosteronism specifically induce chronic kidney disease, in this model, we demonstrated that chronic high-salt diets induced hypertension in this mouse line and resulted in kidney inflammation and injury. Both the aldosterone synthase inhibitor and the MR antagonist markedly blocked high-salt-diet-mediated kidney injury. Thus, this transgenic mouse line can be used to study the pathogenic mechanisms underlying aldosterone and its receptor and to screen therapeutic compounds for aldosterone-mediated hypertension and related complications, such as kidney disease, in humans.

## Simple Summary

Hypertension is a global health issue, often linked to serious complications including kidney disease. Aldosterone, a hormone involved in regulating blood pressure, and its receptor, the mineralocorticoid receptor (MR), play a key role in the pathogenesis of hypertension and related complications. However, there is no well-characterized model for studies to use to better understand their relationships. In this study, we clearly showed that elevated plasma aldosterone levels and salt-induced hypertension caused kidney inflammation and injury in our newly established transgenic mouse model carrying the human aldosterone synthase gene. Both the aldosterone synthase inhibitor and MR antagonist markedly blocked high-salt-diet-induced long-term hypertension and kidney injury. This model therefore offers a valuable tool for studying the pathogenic mechanisms underlying aldosterone and its receptor-mediated hypertension and complications such as kidney disease and for screening therapeutic agents for these symptoms and complications in humans.

## 1. Introduction

Aldosterone is a principal mineralocorticoid hormone secreted from the adrenal cortex. It regulates the body’s electrolyte and water balance and plays a pivotal role in the renin–angiotensin–aldosterone system (RAAS) for blood pressure regulation [1]. Aldosterone acts via both mineralocorticoid receptor (MR)-dependent (genomic) and MR-independent (nongenomic) mechanisms [2,3]. Elevated levels of aldosterone are clearly associated with the development of secondary forms of hypertension [4] as well as essential hypertension [5]. Both experimental and clinical studies indicate that aldosterone is not only linked to hypertension but also promotes brain [6], renal, cardiac, and vasculature [2,7,8,9,10] injury via the upregulation of inflammatory biomarkers [11,12,13,14]. In primary aldosteronism (PA), the adrenal gland releases too much aldosterone, and this results in hypertension as well as end-organ damage such as fibrosis of the heart and kidney [15]. These pathological outcomes can be mitigated with MR antagonist (MRA) treatments [16]. MRAs such as angiotensin-converting enzyme inhibitors (ACEIs) and angiotensin receptor blockers (ARBs) are the current clinical therapies used to antagonize the effects of aldosterone in patients [17,18]. MRAs such as spironolactone and eplerenone completely block the biding of aldosterone to MRs, thereby limiting its effect. However, the use of MRAs is limited by their adverse effects such as electrolyte imbalances [19,20] and reactive increases in circulating aldosterone levels [2,21,22,23]. Additionally, chronic ACEI therapy also stimulates aldosterone release [24]. MRA and ACEI therapies are often associated with increased salt and water retention due to “aldosterone escape” [25,26]. Given the role that aldosterone plays in causing hypertension and promoting cardiovascular and kidney disease, the direct blockade of aldosterone synthesis by inhibiting aldosterone synthase (AS), a specific enzyme that is involved in the generation of aldosterone, may represent a novel but effective alternative approach compared to treatments with MRAs. AS inhibitors (ASIs) attenuate the production of aldosterone directly by blocking the stimulatory effects of angiotensin [27], and in contrast to MRAs, they have additional potential beneficial effects on metabolic parameters in type 2 diabetes [28]. Currently, while the effectiveness of MRA treatments remains to be determined [29] and ASIs are still under development, the underlying genetic and molecular bases of PA and aldosterone are still largely unknown. Therefore, the establishment of proper animal models that can address the current knowledge gap to advance this field becomes urgent and necessary.

While several mouse aldosterone models have been established, they have not been successful in inducing aldosterone-dependent hypertension. In a recent publication, Mopidevi et al. demonstrated that an intron conversion in the AS gene in a knock-in transgenic mouse line increased plasma aldosterone levels by approximately 100% compared to WT mice and 40% compared to the WT polymorphism [30]. However, while the authors demonstrated a 6-week salt-induced blood pressure increase, no further information regarding ASIs, MRAs, or end-organ pathology was available. Another mouse line carries a mutation in the chloride channel ClC-2 gene [31]. This model showed moderately increased plasma aldosterone and salt-induced blood pressure levels but no end-organ damage. Thus, it is necessary to introduce a mouse model with aldosterone-induced end-organ pathology. 

We established a transgenic mouse line carrying the human AS gene (cyp11B2) under the control of the cyp11B1 promoter (Tg-hAS) [32]. In this paper, we demonstrate significantly high plasma levels of human aldosterone in hemizygotes (hAS+/−) and that high-salt diets markedly increase blood pressure. FAD286, a commercial ASI [32], was able to ameliorate this salt-induced hypertension. However, it remains to be determined whether this model is able to develop long-term hypertension and complications like those in humans with high plasma aldosterone concentrations. This model also currently cannot answer whether MRAs or ASIs can chronically ameliorate salt-induced hypertension.

In this study, since female Tg-hAS mice have consistently high plasma aldosterone levels compared to males, we treated Tg-hAS female mice with high-salt diets daily for three months and demonstrated that salt diets persistently induced hypertension in this model. Three-month hypertension in these mice resulted in kidney inflammation and altered kidney function through a markedly increased glomerular infiltration of macrophages [33] and urine albumin–creatinine ratio (uACR). High-salt-induced hypertension was significantly mitigated using either an ASI that significantly inhibited the plasma level of human aldosterone or a clinically relevant MRA in this model. Consistent with hypertension data, both the ASI and ARB significantly attenuated the uACR, kidney inflammation, and glomerular collagen IV, a kidney injury biomarker [14,15,16,17,18], in high-salt-treated female Tg-hAS mice. Our data suggest that this transgenic AS mouse model is a suitable model for studying the pathogenic mechanisms underlying aldosterone and MR-dependent hypertension-mediated kidney disorders, and it can be used to screen potential therapeutic compounds for treatment.

## 2. Materials and Methods

### 2.1. Animals and Treatments

Tg-hAS heterozygous mice were established in our lab [32] and bred in the laboratory of Animal Center at Indiana University School of Medicine. All studies were carried out in accordance with and under the approval of the Indiana University Institutional Animal Care and Use Committee (22092, 8 December 2022). The mice were housed 3–5 per cage, provided unrestricted access to food and water, and kept in a facility with a 12 h light–dark cycle.

Tg-hAS and wild-type (WT) mice from the same litters at 4 weeks old were orally administered with either 0.5% salt control diets (NS) or 4% salt diets (HS) for 12 weeks. After 4 weeks of high-salt treatment, the mice were orally administered 4 mg/kg FAD286 or 100 mg/kg spironolactone (SPL) for an additional 8 weeks. At the end of dose administration, the mice were injected with ketamine–xylazine (75:10 mg/mL, 1 mL/kg body weight) prior to termination. Whole blood was collected from each animal and centrifuged for 15 min at 1500× *g* to separate plasma and blood cells. Following euthanasia, the kidneys and adrenal glands were promptly collected and frozen for further experimental analysis.

### 2.2. BP Measurements

A CODA tail-cuff non-invasive blood pressure system (Model CODA6, Kent Scientific, Torrington, CT, USA) was used to measure the mean systolic pressures (SBPs) and diastolic blood pressures (DBPs) of conscious mice following the manufacturer’s specifications. Prior to measurement, the animals were acclimated to both the environment and the equipment daily for one week. The mice were placed on a warmed pad to ensure comfort during the procedure. An inflatable occlusion cuff was positioned around the tail, along with a volume-pressure recording cuff, following the manufacturer’s guidelines to measure arterial systolic pressure, arterial diastolic pressure, and heart rate. The blood pressure of trained mice was monitored starting around 2 p.m. for 30–40 min, and the mean value of 5 final readings was obtained after 15 initial consecutive readings, whose values were within 5% of the mean. Basal blood pressure was measured in mice fed a normal diet containing 0.5% NaCl (NS, Envigo, Indianapolis, IN, USA) and in mice switched to a high-salt diet (HS, high-salt chow containing 4% NaCl, Envigo, 2019) starting at 4 weeks old. All mice had ad libitum access to tap water throughout this study [32].

### 2.3. Analysis of Blood and Urine

Aldosterone levels in plasms were measured using aldosterone ELISA kit (ab136933, Abcam, Cambridge, MA, USA) following manufacturer’s instructions. uACRs of mouse spot urine were measured to detect leaked protein from injured kidneys. Albumin level in urine was measured using mouse albumin ELISA kit (ab108792, Abcam, Cambridge, MA, USA), and urine creatinine level was measured using creatinine parameter assay kit (KGE005, R&D systems, Minneapolis, MN, USA). uACR was calculated to detect kidney injury. Mouse plasma levels of Na^+^ and K^+^ were measured at Antech Diagnostics (Indianapolis, IN, USA).

### 2.4. The Assessment of Macrophage Activation and Collagen IV Within the Kidneys

Kidney cryosections were incubated with rabbit anti-ionized calcium-binding adapter molecule 1 antibody (Iba1, 1:2000, Abcam, Cambridge, MA, USA) or the collagen IV antibody (1:80, Invitrogen Waltham, MA, USA) followed by an anti-rabbit FITC antibody (1:1000, Abcam, Cambridge, UK) and were visualized under a fluorescence microscope [34]. Sections were scored at 200× magnification (one high-power field, HPF). The average Fluorescence Intensity for ten HPFs was measured using ImageJ 1.54 (Image Processing and Analysis in Java; National Institutes of Health, Bethesda, MD, USA). All assessments were performed by an observer blind to this experiment. 

### 2.5. Statistical Analysis 

One-way analysis of variance (ANOVA) with post hoc comparisons using Dunnett’s test was used for statistical analyses of differences between groups. All data are expressed as mean ± SD. Differences between two means were considered significant when *p* value was less than 0.05.

## 3. Results

### 3.1. The Blood Pressure of Tg-hAS Mice Treated with High Salt in the Presence or Absence of an Aldosterone Inhibitor, FAD286, or an MR Antagonist, SPL 

**Figure 1:**
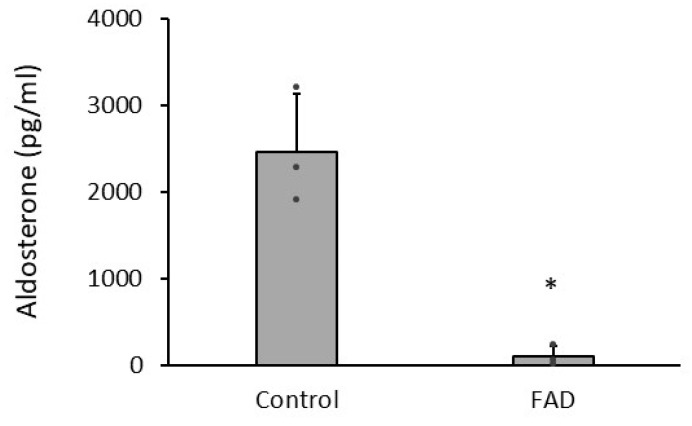
Plasma levels of aldosterone in NS-fed female Tg-hAS mice in presence or absence of FAD286. Plasma levels of aldosterone in female Tg-hAS mice with NS treatment with or without FAD286 were detected by ELISA. Data are presented as mean ± SD, *n* = 6/group. * *p* < 0.05. FAD: FAD286.

**Figure 2:**
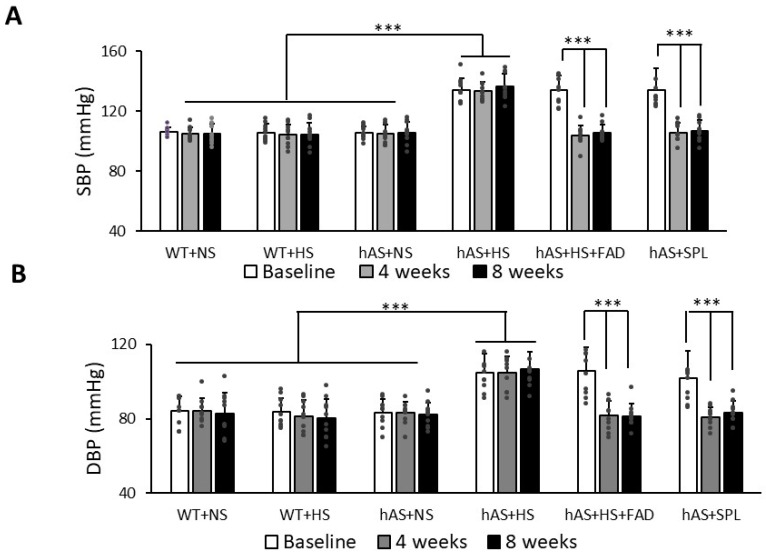
The blood pressure of Tg-hAS mice treated with high salt in the presence or absence of FAD286 or spironolactone. (**A**): SBP, (**B**): DBP. Female WT and Tg-hAS transgenic mice were fed with HS for 1 month, and their BPs were measured (Baseline BPs). The mice on HS were treated continually with FAD286 (FAD) or spironolactone (SPL) for 2 months. The BPs of Tg-hAS mice were measured at the end of 4 weeks and 8 weeks of treatment of FAD or SPL, respectively. Data are presented as the mean ± SD, *n* = 10/group. *** *p* < 0.001. SBP: systolic blood pressure; DBP: diastolic blood pressure; FAD: FAD286; SPL: spironolactone; WT: wild type; hAS: Tg-hAS; NS: normal salt; HS: high salt.

Female mice were orally administered normal-salt (NS) diets mixed with FAD286. After 8 h, plasma aldosterone levels in female Tg-hAS mice were 2464.1 ± 662.8 pg/mL, and FAD286 markedly reduced the plasma level of aldosterone to 99.1 ± 118.7 pg/mL (*p* < 0.001, Figure 1). HS diets augmented the BPs of Tg-hAS mice, as reported in our previous studies [32]. Blood pressures were measured prior to treatment with compound (1-month HS diet) and after treatments with or without 4 mg/kg FAD286 or 50 mg/kg SPL for 8 weeks. Both systolic blood pressures (SBPs) and diastolic blood pressures (DBPs) in 1-month HS-treated Tg-hAS mice were significantly increased compared with NS-treated hAS mice (SBP: 134.1 ± 8.0 mmHg vs. 105.5 ± 4.5 mmHg; DBP: 104.7 ± 10.4 mmHg vs. 83.4 ± 7.2 mmHg, *p* < 0.001) (Figure 2, *n* = 10). In contrast, the HS diets did not affect the BPs of WT mice (SBP: 105.7 ± 5.6 mmHg vs. 106.3 ± 3.0 mmHg; DBP: 83.5 ± 7.7 mmHg vs. 84.4 ± 7.5, mmHg, *p* > 0.05) (Figure 2, *n* = 10). As expected, both FAD286 and SPL markedly reduced mouse SBP and DBP induced by HS after 4 weeks (SBP: FAD286: 103.4 ± 6.6 mmHg, SPL: 105.7 ± 6.1 mmHg vs. 133.2 ± 5.9 mmHg; DBP: FAD286: 81.6 ± 7.9 mmHg, SPL: 80.9 ± 5.0 mmHg vs. 104.8 ± 9.0 mmHg, *p* < 0.001) and 8 weeks of treatment (SBP: FAD286: 105.3 ± 5.5 mmHg, SPL: 106.8 ± 6.9 mmHg vs. 136.4 ± 8.4 mmHg; DBP: FAD286: 81.4 ± 6.5 mmHg, SPL: 83.1 ± 6.3 mmHg vs. 106.5 ± 9.3 mmHg, *p* < 0.001) (Figure 2, *n* = 10). The reduced degrees of BPs in the FAD285-treated group are similar to those in SPL-treated mice. 

### 3.2. uACR and Electrolytes in Tg-hAS Mice Treated with High Salt in Presence or Absence of FAD286 or SPL

**Figure 3:**
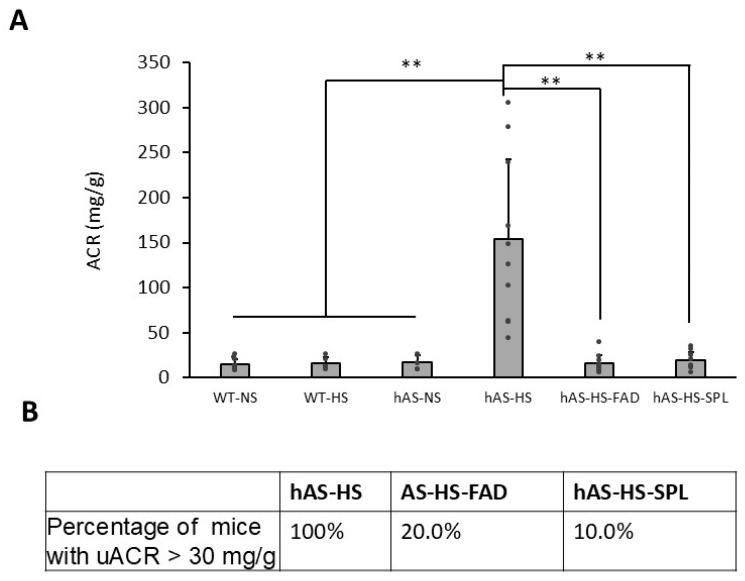
The uACR in Tg-hAS mice treated with high salt in the presence or absence of FAD286 or spironolactone. The 1-month HS-fed female WT and Tg-hAS transgenic mice were treated with or without FAD286 or SPL for 2 months, and the uACRs of mice were measured. (**A**) The percentage of mice with a uACR > 30 mg/g (**B**). Data are presented as the mean ± SD, *n* = 5–10, ** *p* < 0.01. FAD: FAD286; SPL: spironolactone; WT: wild type; hAS: Tg-hAS; NS: normal salt; HS: high salt. ACR: urine-albumin-to-creatinine ratio.

The ratio of urine albumin (mcg/L) to creatinine (mg/L), uACR, is clinically used to determine kidney damage in humans, with the normal range being less than 30 mg/g [35]. In this study, 3-month treatments with HS diets markedly increased the average uACR in Tg-hAS mice from 17.2 ± 7.4 mg/g (*n* = 5) to 153.9 ± 88.5 mg/g (*n* = 10, Figure 3, *p* < 0.01). As expected, both FAD286 and SPL significantly reduced mouse uACR levels to 15.8 ± 9.5 mg/g (n = 10, *p* < 0.01) and 19.5 ± 9.3 mg/g (*n* = 10, *p* < 0.01), respectively (Figure 3A). Interestingly, among the HS-treated Tg-hAS mice (n = 10), the uACRs in all were abnormally above 30, with one even above 300 mg/g. This contrasts with the normal uACRs (<30 mg/g) in wild-type mice fed with either NS (*n* = 5) or HS (*n* = 5). FAD286 and SPL consistently decreased the number of HS-treated Tg-hAS mice with a uACR > 30 mg/g (FAD286, 2 out of 10, 20.0%; SPL, 1 out of 10, 10.0%) (Figure 3B). After treatment with HS for 3 months, the plasma levels of Na^+^ and K^+^ in both WT and Tg-hAS mice were measured at Antech Diagnostics (Indianapolis, IN, USA) (Table 1) to evaluate electrolyte changes in Tg-hAS mice. As previously reported [32], the plasma levels of Na^+^ were significantly higher in Tg-hAS mice as compared to WT mice (154.0 ± 3.13 vs. 150.1 ± 2.69, *n* = 3, *p* < 0.05). The 2-month treatment with FAD markedly reduced Na^+^ levels in Tg-hAS mice to 151.3 ± 2.49 (*p* < 0.05). SPL also decreased Na^+^ levels but without a statistical difference between the SPL-treated mice and the HS-only group (152.1 ± 3.78, *p* > 0.05). In contrast, the plasma levels of K^+^ were significantly lower in Tg-hAS mice compared to WT mice following HS treatments (4.4 ± 0.49 vs. 4.8 ± 0.45, *n* = 3, *p* < 0.05). FAD and SPL increased the K^+^ levels in Tg-hAS mice, but they were still in the normal range (<5.2 mEq/L). 

### 3.3. Glomerular Macrophage Infiltration and Collagen IV Expression in Tg-hAS Mice Treated with High Salt in the Presence or Absence of FAD286 or SPL 

**Figure 4:**
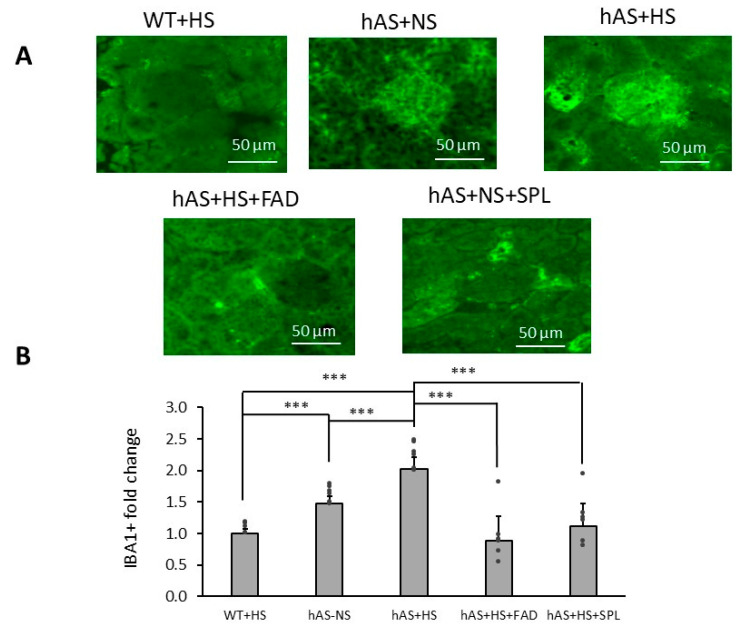
Glomerular macrophage infiltration in Tg-hAS mice treated with high salt in the presence or absence of FAD286 or spironolactone. The 1-month HS-fed Tg-hAS transgenic mice were treated with or without FAD or SPL for 2 months, and macrophage activation in the kidney was measured. (**A**) Representative images of IBA1 staining in the kidney. (**B**) IBA1^+^ immunoreactivity fold change in Tg-hAS mouse kidneys with 2-month treatments of FAD or SPL. Data are presented as the mean ± SD, *n* = 6/group. *** *p* < 0.001. FAD: FAD286; SPL: spironolactone; WT: wild type; hAS: Tg-hAS; NS: normal salt; HS: high salt.

**Figure 5:**
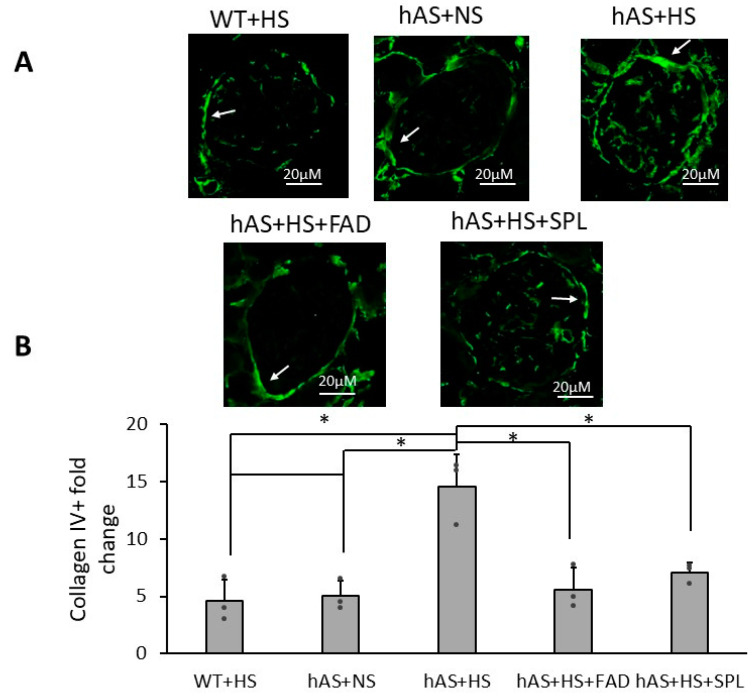
Glomerular collagen IV expression in Tg-hAS mice treated with high salt in the presence or absence of FAD286 or spironolactone. The 1-month HS-fed Tg-hAS transgenic mice were treated with or without FAD or SPL for 2 months, and collagen IV expression in the kidney was measured. (**A**) Representative images of collagen IV staining in the kidney. Arrowheads indicate collagen IV accumulation in the glomerular area. (**B**) Collagen IV immunoreactivity fold change in Tg-hAS mouse kidneys with 2-month treatments of FAD or SPL. Data are presented as the mean ± SD, *n* = 6/group. * *p* < 0.05. FAD: FAD286; SPL: spironolactone; WT: wild type; hAS: Tg-hAS; NS: normal salt; HS: high salt.

Since glomerular macrophage infiltration and collagen IV contribute to hypertension-induced kidney injury and fibrosis [36,37,38], we investigated whether glomerular macrophage infiltration and collagen IV deposition were involved in HS diet-induced kidney injury and whether these could be attenuated by FAD286 and SPL. As expected, Iba1^+^ immunoreactivity was increased two-fold in the glomerular areas of HS-treated Tg-hAS mice compared to HS-treated WT mice (2.03 ± 0.18 vs. 1.00 ± 0.07, *p* < 0.001). Both FAD286 (0.88 ± 0.39) and SPL (1.12 ± 0.36) significantly inhibited HS-induced macrophage infiltration in the kidney glomerular areas of Tg-hAS mice (Figure 4A,B, *p* < 0.001, *n* = 6). Interestingly, Iba1^+^ immunoreactivity in the glomerular areas of NS-treated Tg-hAS mice was increased by 1.47 ± 0.12-fold. Additionally, in the glomerular area, collagen IV accumulation in HS-treated Tg-hAS mice was markedly increased by about 3-fold compared to WT mice (4.6 ± 1.9) (Figure 5A,B, *p* < 0.05, *n* = 3). As expected, both FAD286 (5.6 ± 1.9) and SPL (7.1 ± 0.9) also significantly inhibited HS-induced collagen IV accumulation in kidney glomerular areas (Figure 5A,B, *p* < 0.05, *n* = 3).

## 4. Discussion

This study was designed to further characterize our established hAS+/− mouse model by investigating whether this model could consistently demonstrate salt-induced hypertension, end-organ renal damage that is known to be associated with PA, and changes in kidney function when exposed to ASIs and MRAs. Our goal was to evaluate how this model could be used for future mechanistic and therapeutic studies of aldosterone-mediated disorders. Interestingly, there was a marked difference in plasma aldosterone levels between male and female mice, and hypertension could only be consistently induced by a high-salt diet in females. We found that kidney dysfunction and pathology also only occurred in females, compared to high-salt-fed hypertensive males. As expected, both ASIs and MRAs significantly reduced high-salt-induced hypertension in both sexes and attenuated the 3 mo high-salt diet-induced protein leakage, glomerular inflammation, and glomerular basement membrane collagen IV accumulation in female mouse kidneys. It appeared that salt-induced kidney injury is more aldosterone-dependent after we compared males and females with a similar degree of hypertension. Further studies are required to understand the mechanism behind the alteration of kidney biomarkers such as the glomerular filtration rate, the expression of additional fibrotic and inflammatory markers, and the rationale for the sex differences in kidney pathologies and plasma aldosterone concentrations in hypertensive males. Additionally, further investigations into how high-salt diets induce pathological changes in the cardiovascular system of high-salt-diet-treated hAS+/− are also required. In summary, our data suggest that this transgenic mouse model is suitable to be used for investigating the mechanisms underlying aldosterone-mediated hypertension-dependent disorders such as related chronic kidney diseases and for screening safe and effective aldosterone antagonists for clinical use.

Aldosterone excess has been linked to renal disease development with severe albuminuria, and aldosterone antagonism treatments reduce albumin excretion accompanied by increased serum creatinine [39]. In this transgenic model, 3 mo HS treatments led to a significant number of Tg-hAS mice with abnormally high uACRs (seven out of eight mice) compared to normal ACRs (<30 mg/g), suggesting that hypertensive female Tg-hAS mice induced by HS are ideal models for examining the efficacy of potential protective drugs against aldosterone/salt-associated renal injury. 

Additionally, since aldosterone promotes inflammation, leading to fibrosis and remodeling in the kidney, heart, and vasculature [14] via aldosterone-dependent mechanisms [14,40], and macrophages play a critical role in aldosterone-induced inflammation [41,42], we investigated macrophage infiltration in the kidney glomerulus and found that HS diets specifically induce hypertension-dependent glomerular macrophages in HS-treated Tg-hAS mice. As expected, both ASI and MRA compounds not only reduced salt-induced blood pressures but also attenuated the number of glomerular macrophages. Since female Tg-hAS mice with NS do not have abnormal uACRs or high macrophage infiltration, our data suggest that in this Tg-hAS model, HS-induced hypertension is critical for the observed kidney injury development in female mice. Interestingly, in males with similar ranges of hypertension, we do not observe kidney dysfunction, suggesting that high plasma aldosterone levels are essential to stimulate kidney pathology. A further aging study on end-organ damage in male mice is required. Additionally, distinguishing macrophage subtypes is important in understanding kidney injury and repair [33]. Therefore, further studies are needed on this model to identify what subtypes of macrophages contribute to kidney injury and whether they are targeted by ASIs or MRAs. 

Collagen IV has been widely used in experimental animal studies as a kidney injury biomarker for glomerular sclerosis and interstitial fibrosis [37,38]. We utilized collagen IV alterations as a marker of kidney injury severity in our study. We consistently demonstrated that either FAD286 or MRA SPL can block salt-induced hypertension-dependent glomerular collagen IV overexpression and accumulation in female mice. Further investigations are needed into the role of ASIs and MRAs in kidney, heart, and vasculature fibrosis and remodeling. In the future, this model may be useful in comparing ASIs and MRAs. There is evidence that ASIs may offer additional benefits over MRAs since they directly reduce aldosterone levels and do not promote the activation of NF-κB in neutrophils [14]. 

Hypertension is one of the most important global health challenges due to its high prevalence, morbidity and mortality, and resultant end-organ damage, leading to cardiovascular disease, ischemic and hemorrhagic stroke, and chronic kidney disease [43]. Aldosterone is a principal mineralocorticoid hormone that acts through the RAAS pathway, contributing to hypertension development [2,3]. There is a clear relationship between elevated levels of aldosterone and the development of resistant hypertension [4,5]. In particular, aldosterone appears to play an important role in resistant hypertension, suggesting that targeting the aldosterone pathway may treat diuretic- and therapy-resistant hypertension [44]. Anti-hypertensive aldosterone’s downstream MR blockers such as spironolactone and eplerenone have been used in clinics. However, they are associated with reactive increases in circulating aldosterone levels that theoretically exacerbate the detrimental actions of aldosterone [21,22,23]. Therefore, it is necessary to develop other medicines antagonizing aldosterone such as ASIs to treat aldosterone-related hypertension and its complications. Interestingly, it has been reported that ASIs, in contrast to MRAs, have additional potential beneficial effects on metabolic parameters in type 2 diabetes [28]. Therefore, this transgenic model carrying the human aldosterone synthase gene is an ideal model for developing a novel and effective alternative ASI approach as compared to treatments using MRAs. 

## 5. Conclusions

In this study, we provide important pathogenic insights into our established mouse model for high-salt- and aldosterone-mediated hypertension. Our data suggest that this mouse model has biologically relevant utility for investigating the molecular mechanisms underlying aldosterone/hypertension-mediated pathogenesis and developing treatments for these disorders.

## Figures and Tables

**Table 1:** Plasma electrolytes in WT and Tg-hAS mice treated with high salt in the presence or absence of FAD286 or spironolactone. Data are presented as the mean ± SD. * *p* < 0.05, Tg-hAS mice with HS versus WT mice with HS. # *p* < 0.05, Tg-hAS mice with HS and FAD or SPL treatment versus Tg-hAS mice with HS. FAD: FAD286; SPL: spironolactone; WT: wild type; hAS: Tg-hAS; HS: high salt.

Groups	Sodium (mEq/L)	Potassium (mEq/L)
WT + HS	150.1 ± 2.69	4.8 ± 0.45
hAS + HS	154.0 ± 3.13 *	4.4 ±0.49 *
hAS + HS + FAD	151.3 ± 2.49 ^#^	5.0 ± 0.73 ^#^
hAS + HS + SPL	152.1 ± 3.78	4.7 ± 0.56

## Data Availability

Data are contained within the article.

## References

[1] Kanugula A.K., Kaur J., Batra J., Ankireddypalli A.R., Velagapudi R. (2023). Renin-Angiotensin System: Updated Understanding and Role in Physiological and Pathophysiological States. Cureus.

[2] Brown N.J. (2013). Contribution of aldosterone to cardiovascular and renal inflammation and fibrosis. Nat. Rev. Nephrol..

[3] Nguyen Dinh Cat A., Jaisser F. (2012). Extrarenal effects of aldosterone. Curr. Opin. Nephrol. Hypertens..

[4] Conn J.W. (1955). Primary aldosteronism. J. Lab. Clin. Med..

[5] Genest J., Lemieux G., Davignon A., Korw E., Nowaczynski W., Steyermark P. (1956). Human arterial hypertension: A state of mild chronic hyperaldosteronism?. Science.

[6] Lin X., Ullah M.H.E., Wu X., Xu F., Shan S.-K., Lei L.-M., Yuan L.-Q., Liu J. (2021). Cerebro-Cardiovascular Risk, Target Organ Damage, and Treatment Outcomes in Primary Aldosteronism. Front. Cardiovasc. Med..

[7] Anavekar N.S., Solomon S.D. (2005). Angiotensin II receptor blockade and ventricular remodelling. J. Renin Angiotensin Aldosterone Syst..

[8] Pu Q., Neves M.F., Virdis A., Touyz R.M., Schiffrin E.L. (2003). Endothelin antagonism on aldosterone-induced oxidative stress and vascular remodeling. Hypertension.

[9] Rocha R., Rudolph A.E., Frierdich G.E., Nachowiak D.A., Kekec B.K., Blomme E.A.G., McMahon E.G., Delyani J.A. (2002). Aldosterone induces a vascular inflammatory phenotype in the rat heart. Am. J. Physiol. Heart Circ. Physiol..

[10] Namsolleck P., Unger T. (2014). Aldosterone synthase inhibitors in cardiovascular and renal diseases. Nephrol. Dial. Transplant..

[11] Brown N.J. (2008). Aldosterone and vascular inflammation. Hypertension.

[12] Moraes L.A., Paul-Clark M.J., Rickman A., Flower R.J., Goulding N.J., Perretti M. (2005). Ligand-specific glucocorticoid receptor activation in human platelets. Blood.

[13] Martin-Fernandez B., Rubio-Navarro A., Cortegano I., Ballesteros S., Alía M., Cannata-Ortiz P., Olivares-Álvaro E., Egido J., de Andrés B., Gaspar M.L. (2016). Aldosterone Induces Renal Fibrosis and Inflammatory M1-Macrophage Subtype via Mineralocorticoid Receptor in Rats. PLoS ONE.

[14] Gilbert K.C., Brown N.J. (2010). Aldosterone and inflammation. Curr. Opin. Endocrinol. Diabetes Obes..

[15] Vaidya A., Mulatero P., Baudrand R., Adler G.K. (2018). The Expanding Spectrum of Primary Aldosteronism: Implications for Diagnosis, Pathogenesis, and Treatment. Endocr. Rev..

[16] Funder J.W., Carey R.M., Mantero F., Murad M.H., Reincke M., Shibata H., Stowasser M., Young W.F. (2016). The Management of Primary Aldosteronism: Case Detection, Diagnosis, and Treatment: An Endocrine Society Clinical Practice Guideline. J. Clin. Endocrinol. Metab..

[17] Liu L.C., Schutte E., Gansevoort R.T., van der Meer P., Voors A.A. (2015). Finerenone : Third-generation mineralocorticoid receptor antagonist for the treatment of heart failure and diabetic kidney disease. Expert Opin. Investig. Drugs.

[18] Pitt B., Williams G., Remme W., Martinez F., Lopez-Sendon J., Zannad F., Neaton J., Roniker B., Hurley S., Burns D. (2001). The EPHESUS trial: Eplerenone in patients with heart failure due to systolic dysfunction complicating acute myocardial infarction. Eplerenone Post-AMI Heart Failure Efficacy and Survival Study. Cardiovasc. Drugs Ther..

[19] Roscioni S.S., de Zeeuw D., Bakker S.J.L., Heerspink H.J.L. (2012). Management of hyperkalaemia consequent to mineralocorticoid-receptor antagonist therapy. Nat. Rev. Nephrol..

[20] Shavit L., Lifschitz M.D., Epstein M. (2012). Aldosterone blockade and the mineralocorticoid receptor in the management of chronic kidney disease: Current concepts and emerging treatment paradigms. Kidney Int..

[21] Grossmann C., Gekle M. (2009). New aspects of rapid aldosterone signaling. Mol. Cell. Endocrinol..

[22] Good D.W. (2007). Nongenomic actions of aldosterone on the renal tubule. Hypertension.

[23] Mihailidou A.S., Funder J.W. (2005). Nongenomic effects of mineralocorticoid receptor activation in the cardiovascular system. Steroids.

[24] MacFadyen R.J., Lee A.F.C., Morton J.J., Pringle S.D., Struthers A.D. (1999). How often are angiotensin II and aldosterone concentrations raised during chronic ACE inhibitor treatment in cardiac failure?. Heart.

[25] McKelvie R.S., Yusuf S., Pericak D., Avezum A., Burns R.J., Probstfield J., Tsuyuki R.T., White M., Rouleau J., Latini R. (1999). Comparison of candesartan, enalapril, and their combination in congestive heart failure: Randomized evaluation of strategies for left ventricular dysfunction (RESOLVD) pilot study. The RESOLVD Pilot Study Investigators. Circulation.

[26] Bomback A.S., Klemmer P.J. (2007). The incidence and implications of aldosterone breakthrough. Nat. Clin. Pract. Nephrol..

[27] Schiffrin E.L. (2006). Effects of aldosterone on the vasculature. Hypertension.

[28] Hofmann A., Brunssen C., Peitzsch M., Martin M., Mittag J., Jannasch A., Engelmann F., Brown N.F., Weldon S.M., Huber J. (2016). Aldosterone Synthase Inhibition Improves Glucose Tolerance in Zucker Diabetic Fatty (ZDF) Rats. Endocrinology.

[29] Hundemer G.L., Curhan G.C., Yozamp N., Wang M., Vaidya A. (2018). Cardiometabolic outcomes and mortality in medically treated primary aldosteronism: A retrospective cohort study. Lancet Diabetes Endocrinol..

[30] Mopidevi B., Sivankutty I., Hao S., Ferreri N.R., Kumar A. (2020). Effects of intron conversion in the human CYP11B2 gene on its transcription and blood pressure regulation in transgenic mice. J. Biol. Chem..

[31] Schewe J., Seidel E., Forslund S., Marko L., Peters J., Muller D.N., Fahlke C., Stölting G., Scholl U. (2022). Author Correction: Elevated aldosterone and blood pressure in a mouse model of familial hyperaldosteronism with ClC-2 mutation. Nat. Commun..

[32] Gu H., Ma Z., Wang J., Zhu T., Du N., Shatara A., Yi X., Kowala M.C., Du Y. (2017). Salt-dependent Blood Pressure in Human Aldosterone Synthase-Transgenic Mice. Sci. Rep..

[33] Huen S.C., Cantley L.G. (2017). Macrophages in Renal Injury and Repair. Annu. Rev. Physiol..

[34] Gu H., Kirchhein Y., Zhu T., Zhao G., Peng H., Du E., Liu J., Mastrianni J.A., Farlow M.R., Dodel R. (2019). IVIG Delays Onset in a Mouse Model of Gerstmann-Straussler-Scheinker Disease. Mol. Neurobiol..

[35] Zhang A., Li M., Qiu J., Sun J., Su Y., Cai S., Bao Q., Cheng B., Ma S., Zhang Y. (2022). The relationship between urinary albumin to creatinine ratio and all-cause mortality in the elderly population in the Chinese community: A 10-year follow-up study. BMC Nephrol..

[36] Wen Y., Crowley S.D. (2020). The varying roles of macrophages in kidney injury and repair. Curr. Opin. Nephrol. Hypertens..

[37] Boor P., Konieczny A., Villa L., Kunter U., van Roeyen C.R., LaRochelle W.J., Smithson G., Arrol S., Ostendorf T., Floege J. (2007). PDGF-D inhibition by CR002 ameliorates tubulointerstitial fibrosis following experimental glomerulonephritis. Nephrol. Dial. Transplant..

[38] Boor P., Celec P., Behuliak M., Grančič P., Kebis A., Kukan M., Pronayová N., Liptaj T., Ostendorf T., Šebeková K. (2009). Regular moderate exercise reduces advanced glycation and ameliorates early diabetic nephropathy in obese Zucker rats. Metabolism.

[39] Monticone S., Sconfienza E., D’Ascenzo F., Buffolo F., Satoh F., Sechi L.A., Veglio F., Mulatero P. (2020). Renal damage in primary aldosteronism: A systematic review and meta-analysis. J. Hypertens..

[40] Boldyreff B., Wehling M. (2004). Aldosterone: Refreshing a slow hormone by swift action. News Physiol. Sci..

[41] Leibovitz E., Ebrahimian T., Paradis P., Schiffrin E.L. (2009). Aldosterone induces arterial stiffness in absence of oxidative stress and endothelial dysfunction. J. Hypertens..

[42] Rickard A.J., Morgan J., Tesch G., Funder J.W., Fuller P.J., Young M.J. (2009). Deletion of mineralocorticoid receptors from macrophages protects against deoxycorticosterone/salt-induced cardiac fibrosis and increased blood pressure. Hypertension.

[43] Vasan R.S., Larson M.G., Leip E.P., Evans J.C., O’Donnell C.J., Kannel W.B., Levy D. (2001). Impact of high-normal blood pressure on the risk of cardiovascular disease. N. Engl. J. Med..

[44] Stevens T.M., Saha J., Du Y. (2018). The Role of Aldosterone in Hypertension and Related Morbidities. Ann. Hypertens..

